# Dihydroartemisinin is a Hypoxia-Active Anti-Cancer Drug in Colorectal Carcinoma Cells

**DOI:** 10.3389/fonc.2014.00116

**Published:** 2014-05-19

**Authors:** Teona Ontikatze, Justine Rudner, René Handrick, Claus Belka, Verena Jendrossek

**Affiliations:** ^1^Institute of Cell Biology (Cancer Research), Faculty of Medicine, University of Duisburg-Essen, Essen, Germany; ^2^Institute of Applied Biotechnology, University of Applied Sciences, Biberach, Germany; ^3^Department of Radiation Oncology, Ludwig-Maximilian University Munich, Munich, Germany

**Keywords:** therapy resistance, hypoxia, dihydroartemisinin, apoptosis, autophagy, Bim, Puma, BNIP3

## Abstract

Tumor hypoxia is one main biological factor that drives resistance to chemotherapy and radiotherapy. To develop a novel strategy for overcoming hypoxia-induced therapy resistance, we examined the anti-neoplastic activity of the reactive oxygen donor dihydroartemisinin (DHA) in human colon cancer cell lines in normoxia and severe hypoxia. In addition, we analyzed the involvement of the intrinsic apoptosis pathway for DHA-mediated cytotoxicity in HCT116 cells in short-term and long-term *in vitro* assays. When applied at lower concentrations (≤25 μM), DHA induced apoptosis in Colo205, HCT15, and HCT116 cells, whereas necrotic cell death was increased when cells were treated with higher DHA concentrations (50 μM). However, no preference for DHA-induced apoptosis or necrosis could be detected between the treatment under normoxic or hypoxic conditions. Moreover, DHA potently reduced clonogenic survival of HCT116 cells in normoxia and hypoxia. Treatment of HCT116 cells with 25 μM DHA resulted in activation of Bax under normoxic and hypoxic conditions. Interestingly, cytochrome *c* release from the mitochondria and caspase-activation were observed only under normoxic conditions, whereas, under hypoxic conditions DHA induced a caspase-independent apoptosis-like cell death. However, under both conditions, generation of reactive oxygen species was an important mediator of DHA-induced toxicity. Further molecular analysis suggests that DHA-mediated cell death involves different sets of pro-apoptotic Bcl-2 family members. The pronounced cytotoxic activity of DHA in severe hypoxia as well as normoxia offers new perspectives for targeting the hypoxic tumor cell fraction to improve treatment outcome for cancer patients.

## Introduction

Tumor cell resistance to classical chemotherapy and radiotherapy remains a major obstacle in the treatment of solid human tumors (1). Genetic or epigenetic alterations in the tumor cells that affect cellular death and survival signaling can allow the tumor cells to escape the cytotoxic action of standard genotoxic therapies and molecularly targeted agents. Moreover, specific conditions within the tumor microenvironment and the intimate dialog between tumor cells and their surrounding stroma by soluble growth- and survival-promoting factors provide a multitude of mechanisms for therapy resistance. Therefore, current research activities focus on the identification of drugs that are active under adverse environmental conditions to counteract environment-mediated resistance to therapies.

Tumor hypoxia is a common characteristic of solid human tumors and is mostly associated with poor prognosis (2). Tumor hypoxia plays a central role in tumor progression and is one main biological factor that drives therapy resistance at multiple levels: reduced availability of molecular oxygen during acute hypoxia reduces the burst of reactive oxygen species (ROS) induced by genotoxic treatments, e.g., radiotherapy, and thus hampers the manifestation of DNA-damage and the generation of pro-death signals. Moreover, acute hypoxia also profoundly reorganizes cell signaling to increase the death threshold [overview in Ref. (3)]. Finally, chronic intermittent tumor hypoxia promotes selection of hypoxia-tolerant cells that are characterized by diminished apoptotic potential, increased therapy resistance, and worse prognosis (4–6).

At present, researchers follow diverse approaches to overcome hypoxia-mediated therapy resistance: (i) reduce the hypoxic fraction of the tumor by increasing the blood oxygen tension, (ii) specifically kill hypoxic cells by using bioreductive drugs, and (iii) reduce the tolerance of hypoxic cells by using signal transduction inhibitors targeting pathways that are essential for the survival of hypoxic cells (2, 7).

Here, we propose a novel strategy to overcome therapy resistance under conditions of acute hypoxia by using the cyclic endoperoxide dihydroartemisinin (DHA). DHA belongs to a family of compounds derived from the natural sesquiterpene lactone artemisinin (Coartem^®^/Riamet^®^) that are known to generate ROS-like superoxide anions and hydroxyl radicals as well as carbon-centered radicals upon activation of their endoperoxide bridge in the presence of ferrous iron (8). Artemisinin and DHA are already in clinical use for antimalaria treatment (9). However, both drugs also exert potent anti-neoplastic effects on human tumor cells in preclinical *in vitro* and *in vivo* investigations (10–12). Earlier studies revealed that the generation of ROS and carbon-centered radicals is critical for the toxic effects of artemisinin and derivatives on malaria parasites (13, 14). These reactive molecules also contribute to the potent anti-cancer activity of these compounds through alkylation of essential proteins and induction of oxidative damage to membrane lipids and DNA and subsequent ROS-dependent apoptosis that includes the activation of pro-apoptotic Bcl-2 family member Bax, and caspase-activation (11, 15, 16).

Though anti-neoplastic activity of artemisinin and derivatives is well-documented for standard treatment conditions in normoxia, the potential of these drugs to kill cancer cells under conditions of acute hypoxia and the involved molecular pathways have not yet been studied. On the basis of their potential to generate ROS and further reactive molecular species, we hypothesized that treatment with compounds of the Artemisinin drug family may be a promising approach to efficiently attack hypoxic cancer cells and overcome therapy resistance induced by acute hypoxia. To verify our hypothesis, we compared the anti-neoplastic activity of DHA under normoxic and hypoxic conditions using three different colorectal cancer cell lines as experimental model. We demonstrate for the first time that DHA is a hypoxia-active drug that efficiently kills colon cancer cells even in presence of very low oxygen levels. When treated at lower DHA concentrations (≤25 μM), colon cancer cells mainly underwent apoptosis, whereas necrosis was increased when higher doses of DHA (50 μM) were applied.

Further molecular analysis of DHA-mediated cytotoxicity in HCT116 cells revealed that DHA induced the canonical mitochondrial apoptosis pathway that includes the activation of Bax, cytochrome *c* release from mitochondria into the cytosol, caspase-activation, dissipation of the mitochondrial transmembrane potential (ΔΨm) and DNA-fragmentation. Although Bax-activation occurred to similar extent when HCT116 cells were treated under normoxic conditions, release of cytochrome *c* and caspase-activation were almost abrogated. However, a high amount of cells with fragmented or condensed DNA was observed even in the absence of caspase-activation suggesting the induction of caspase-independent apoptotic cell death by DHA in severely hypoxic cancer cells. Moreover, under both conditions DHA-induced ROS production mediated the cytotoxic effect since blocking the ROS production resulted in reduced DNA-fragmentation. In addition, hypoxic HCT116 cells induced a different set of regulatory BH3-only proteins in response to DHA compared to normoxic cells suggesting that different BH3-only proteins might contribute to the canonical and non-canonical apoptosis in normoxia and hypoxia by inhibiting anti-apoptotic Bcl-2 family members and facilitating the activation of the Bax.

## Materials and Methods

### Chemicals and drugs

Dihydroartemisinin [(3,5,6,8,9,10,12R,12aR)-decahydro-3,6,9- trimethyl-3,12-epoxy-12H-pyrano[4,3-j]-1,2-benzodioxepin-10- ol, C_15_H_24_O_5_)] and propidium iodide (PI) were obtained from Sigma-Aldrich (Deisenhofen, Germany). Hoechst 33342 was purchased from Calbiochem (Bad Soden, Germany). The pan-caspase inhibitor benzyloxycarbonyl-Val-Ala-Asp(OMe)-fluoromethylketone (zVAD-fmk) was obtained from Bachem (Bubendorf, Switzerland). Tetramethylrhodamine ethyl ester perchlorate (TMRE) and dihydroethidium (DHE) were from Molecular Probes (MoBiTec, Goettingen, Germany).

Antibodies specific for full length and cleaved poly (ADP-ribose) polymerase (PARP), caspase-3, light chain 3B (LC3B), Bax, Bak, Bcl-xL, and Puma were obtained from Cell Signaling (Frankfurt, Germany). Bcl-2 antibody was purchased from Santa Cruz Biotechnology (Heidelberg, Germany), Bim antibody was purchased from Epitomics (Biomol, Hamburg, Germany). The antibody specifically recognizing the active conformation of Bax (Bax NT) was from Upstate (Hamburg, Germany). Moreover, we used antibodies specific for Noxa (Calbiochem, Darmstadt, Germany), cytochrome *c* (Pharmingen, Hamburg, Germany), or β-actin (Sigma-Aldrich, Deisenhofen, Germany) as well as HRP-conjugated and Cy2-conjugated secondary antibodies (Amersham-Biosciences, Freiburg, Germany).

All other chemicals and drugs were from Sigma-Aldrich if not otherwise specified.

### Cell culture

Colon cancer cell lines HCT15, Colo205, and HCT116 were obtained from ATCC (Bethesda, MD, USA). Morphology and phenotype of the distinct cell lines were routinely tested before and during data acquisition.

Cells were grown in RPMI 1640 medium supplemented with 10% (v/v) fetal calf serum (Gibco Life Technologies, Eggenstein, Germany) and maintained in a humidified incubator at 37°C and 5% CO_2_ (normoxic conditions). Hypoxic cells were grown in a humidified hypoxia work station (In vivo 400, Ruskinn Technology Ltd., IUL Instruments GmbH, Königswinter, Germany) at 37°C, 0.2% O_2_, and 5% CO_2_.

### Drug treatment

Cells were treated 24 h after seeding with 0–50 μM DHA. For treatment under hypoxic conditions, cells were transferred to the hypoxic chamber 2 h before drug treatment. For all experiments, 0.1% ethanol was used as solvent control.

### Quantification of cell viability and cell proliferation

The number of living cells was determined upon staining of the cells with the vital dye trypan blue. For this, cells were harvested with Trypsin-EDTA, re-suspended in fresh medium, diluted with trypan blue, and counted employing a Neubauer chamber.

In addition, metabolic activity of the cells was determined as an indirect measure of cell viability using the WST-1 proliferation assay according to the manufacturer’s instructions (Roche Applied Science, Mannheim, Germany). Reduction of the water-soluble tetrazolium salt WST-1 to formazan was determined using an ELISA reader (Bio-Tek, Bad Friedrichshall, Germany; 480 and 680 nm).

### Colony formation assay

For this long-term assay, 200–1600 cells/well were plated in 6-well plates, incubated in normoxia for 24 h, and then treated with DHA in normoxia or severe hypoxia. Plates seeded for treatment under hypoxic conditions were transferred into the hypoxic chamber 2 h prior to DHA treatment until 24 h after treatment and subsequently incubated in normoxia. Plates were incubated for a total of 10 days to allow growth of single colonies. Cells were then fixed in 3.7% formaldehyde and 70% ethanol and subsequently stained with 0.05% Coomassie Brilliant Blue. Colonies (≥50 cells/colony) were counted under the microscope at fivefold magnification. The survival curves were established by plotting the log of the surviving fraction against the treatment dose. Fitting of the curves was performed using Excel software.

### Analysis of apoptotic and necrotic cell death by fluorescence microscopy

Changes in nuclear morphology indicative for apoptosis and necrosis were analyzed by fluorescence microscopy (Zeiss Axiovert 200, Carl Zeiss, Göttingen, Germany; G365/FT395/LP420 filter set) upon cell staining with 1.5 μM Hoechst 33342 and 2.5 μg/ml PI. Apoptotic and necrotic cells were quantified by counting the relative amount of fragmented blue or red nuclei (early or late apoptosis, respectively) and non-fragmented red nuclei (necrosis). At least 100 cells were quantified in three independent fields per well.

### Flow cytometry analyses

For quantification of apoptotic DNA-fragmentation (sub-G1 population), cells were incubated for 60 min with a staining solution containing 0.1% (w/v) sodium citrate, 50 μg/ml PI, and 0.05% (v/v) Triton X-100 (v/v) and subsequently analyzed by flow cytometry (FACS Calibur, Becton Dickinson, Heidelberg, Germany; FL-2).

Dissipation of the ΔΨm was measured using the ΔΨm-specific dye TMRE (FL-2) as described elsewhere (17). To ensure a complete dissipation of ΔΨm, cells were treated with 100 μM protonophore carbonyl cyanide m-chlorophenylhydrazone (CCCP) before incubation with TMRE.

To quantify ROS production, cells were stained for 15 min at 37°C with 5 μM of the ROS-sensitive dye DHE (Molecular Probes, MoBiTec, Göttingen, Germany) and washed subsequently once with PBS. ROS-positive cells were detected by flow cytometry (FL-2). Treatment with 250 μM H_2_O_2_ (2 h) was used as positive control for ROS formation. As additional control, cells were pretreated with 2 mM *N*-acetylcysteine (Acc) to block ROS formation.

Activation of Bax was determined using the activation-specific anti-Bax NT antibody. In brief, cells were fixed for 20 min on ice in 2.5% (w/v) PFA/PBS, washed in 1% (v/v) FCS/PBS, permeabilized for 30 min on ice with 0.1% (v/v) Triton X-100/PBS, washed with PBS, and then incubated for 15 min at room temperature (RT) with a blocking solution (10% FCS/PBS). Cells were stained for 30 min with the activation-specific anti-Bax NT antibody or the respective isotype control, washed, incubated for 30 min with a cy2-conjugated anti-rabbit secondary antibody (Amersham, Freiburg, Germany), washed again, and then suspended in blocking buffer for flow cytometric analysis (FL-1).

### Release of cytochrome *c*

Cells were plated on cover slips, washed with PBS, and fixed for 15 min with 3% (v/v) paraformaldehyde in PBS at RT. Subsequently, cells were permeabilized for 10 min with 0.2% (v/v) Triton X-100 in PBS, washed with PBS, blocked for 30 min in 1% (v/v) fetal calf serum, and subsequently incubated for 1 h at RT with the anti-cytochrome *c* primary antibody (Pharmingen, Becton Dickinson). After repeated washing, cells were incubated for 45 min at RT in the dark with the secondary Cy2-conjugated anti-mouse antibody (Amersham, Freiburg, Germany). Finally, the cover slips were mounted with Fluorescence Mounting Medium (Dako, Hamburg, Germany). Cytochrome *c* release was visualized by fluorescence microscopy using a Zeiss Axiovert 200 microscope equipped with an Apotome with an 63× oil objective and GFP filter set (Carl Zeiss, Jena, Germany). Analysis of green fluorescence and overlay were performed with AxioVision software (Carl Zeiss, Jena, Germany).

### Western blot analysis

Cells were lysed for 10 min at 99°C in 62.5 mM Tris-HCl (pH 6.8), 2% (w/v) SDS, 10% (v/v) glycerol, 50 mM dithiothreitol, and 0.01% (w/v) bromophenol blue. Proteins were separated by SDS-PAGE and blotted onto PVDF-membranes (Roth, Karlsruhe, Germany). After blocking with 5% (w/v) non-fat dry milk, membranes were incubated at 4°C over night with the respective primary antibody (1:20,000 for β-actin, 1:1000 for all other antibodies). After washing, the membranes were incubated for 1 h at RT with the secondary antibody (anti-IgG-HRP 1:2000, Amersham-Biosciences, Freiburg, Germany), washed again, and developed using enhanced chemiluminescence staining (ECL western blotting analysis system, Amersham-Biosciences, Freiburg, Germany). We indicated that protein levels were quantified by densitometry using ImageJ software (ImageJ 1.40g, NIH, USA). The respective protein levels were normalized to β-actin levels.

### Statistics

Data represent mean values of at least three independent experiments ± standard deviation (SD). Specific values represent normalization to respective solvent controls [% treated cells − % solvent control]. Data analysis was performed by two-tail unpaired *t*-test (Prism5™ software, GraphPad Inc., La Jolla, CA, USA) or two-way ANOVA test using parametric methods and employing Bonferroni multiple comparison post-test where appropriate. *P*-values ≤0.05 were considered as significant.

## Results

### Dihydroartemisinin exerts potent anti-neoplastic effects under normoxic and hypoxic conditions

**Figure 1:**
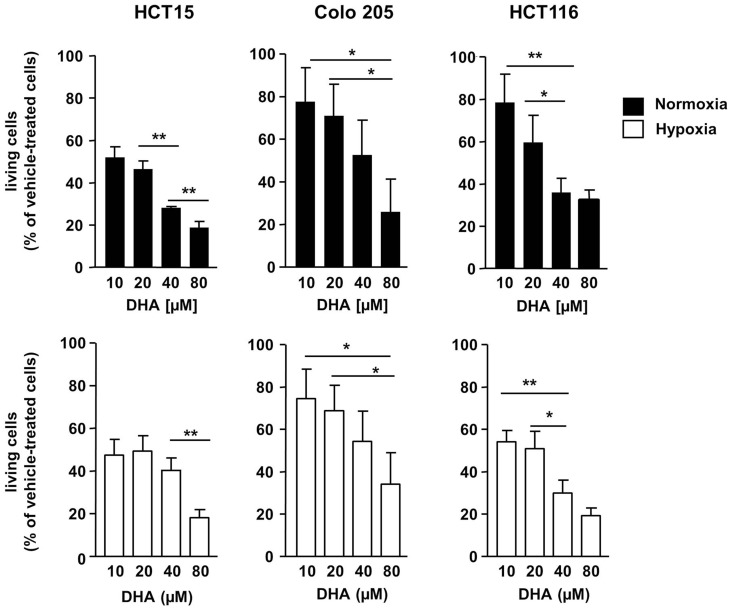
**DHA exerts potent anti-neoplastic effects on colon cancer cells in normoxia and severe hypoxia**. HCT15, Colo205, and HCT116 colon cancer cells were treated for 48 h with various concentrations of DHA (0–80 μM) under normoxic (21% O_2_; black bars) or severely hypoxic conditions (0.2% O_2_; white bars) as indicated. Cell proliferation and survival were monitored 48 h after treatment by using the WST-1 assay that quantifies the metabolic activity of viable cells. Ethanol (0.1%) was used as solvent control. DHA reduces the number of viable HCT15, HCT116, and Colo 205 cells in a concentration-dependent manner in normoxia (upper panel) and hypoxia (lower panel). DHA displays almost similar anti-neoplastic activity in normoxia and hypoxia. Data represent means ± SD (*n* = 3). Values were normalized to the ethanol control. *P*-values were calculated applying unpaired two-tailed *t*-test. *P < 0.05; **P < 0.01; ***P < 0.001. Nx, normoxia; Hx, hypoxia.

To characterize the anti-neoplastic potential of DHA in severely hypoxic cancer cells, we first compared tumor cell survival after treatment with DHA under normoxic and under severely hypoxic conditions. For this, we treated three different human colon cancer cells (HCT15, Colo205, and HCT116 cells) with 0–80 μM DHA for 48 h in the presence of normal (21% O_2_) or greatly reduced oxygen tensions (0.2% O_2_). DHA significantly reduced the number of viable HCT15, HCT116, and Colo 205 colon cancer cells in a concentration-dependent manner under normoxic (Figure 1, upper panel) as well as under hypoxic conditions (Figure 1, lower panel). The response of the three cell lines to DHA differed slightly depending on the drug concentration and the oxygen levels. Whereas HCT15 cells turned out to be highly sensitive in normoxia and hypoxia with almost 50% reduction in the number of viable cells upon treatment with 10 μM DHA under both conditions, the sensitivity of HCT116 cells was higher in hypoxia (almost 50% reduction in the number of viable cells with 10 μM DHA compared to 20% in normoxia). In contrast, the number of viable cells decreased only by around 20% in Colo205 upon treatment with 10 μM DHA under both, normoxic and hypoxic conditions. Nevertheless, the results indicate that DHA is highly active in normoxic and hypoxic colorectal cancer cells.

**Figure 2:**
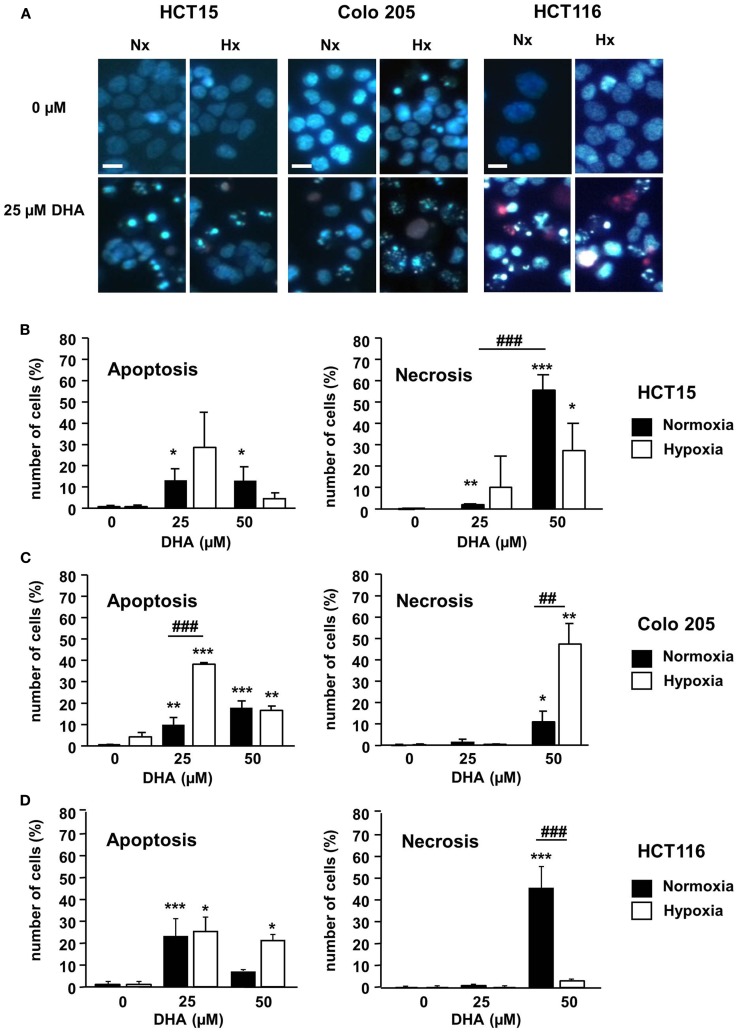
**DHA induces apoptotic and necrotic cell death in colon cancer cells in normoxia and hypoxia**. HCT15, Colo 205, and HCT116 cells were treated for 24 h with solvent (Ethanol, 0 μM DHA) or DHA (25, 50 μM) under normoxic (21% O_2_; black bars) or under severely hypoxic conditions (0.2% O_2_; white bars). To visualize apoptotic and necrotic cell death, cells were stained with Hoechst (blue) and PI (red) and submitted to fluorescence microscopy 24 h post treatment. **(A)** Representative fluorescence microscopy pictures from one of three independent experiments are shown. Scale bar corresponds to 100 μm. Pictures were taken using 10-fold magnification. **(B–D)** At concentrations of 25 μM and below, DHA preferentially induced apoptosis, whereas at 50 μM DHA necrosis became more prominent. The percentage of apoptotic and necrotic cells in **(B)** HCT15 cells, **(C)** Colo 205 cells, and **(D)** HCT116 cells was quantified by counting the fraction of cells with normal, apoptotic, and necrotic nuclei using fluorescence microscopy (DAPI channel). At least 100 cells per field were quantified from three independent areas per condition. Data represent means from three independent experiments ± SD. Statistical analysis was performed according to unpaired two-tailed *t*-test. *P < 0.05; **^, ##^*P* < 0.01, ***^, ###^*P* < 0.001. *Indicates the significance between the solvent treated control cells and DHA-treated cells either in normoxia or hypoxia, ^#^indicates the significance between normoxic and hypoxic cells treated with the same concentration of DHA.

To characterize the mechanism of cytotoxicity more closely, the mode of cell death induced by DHA was analyzed. For this purpose, the three colon cancer cell lines were treated with 0–50 μM DHA under normoxic and hypoxic conditions. Forty-eight hours later, the cells were co-stained with the membrane permeable DNA dye Hoechst 33342 and PI, a DNA dye that cannot enter cells with intact plasma membrane integrity, to distinguish between apoptotic and necrotic cell death (Figure 2A). PI-negative and PI-positive cells with condensed DNA were mainly observed after treatment with 25 μM DHA under normoxia or hypoxia indicating that at this applied concentration the dominant mode of cell death was apoptosis (Figures 2B–D, left panels). In contrast, treatment with 50 μM DHA yielded elevated levels of PI-positive cells without DNA condensation suggesting that necrotic cell death was increased in response to treatment with higher DHA concentrations (Figures 2B–D, right panels). However, the impact of normoxia or hypoxia on DHA-induced necrosis is not clear. Whereas DHA-induced necrosis was much higher in normoxic than in hypoxic HCT15 and HCT116 cells, Colo205 cells showed more necrosis upon DHA treatment in hypoxia compared to normoxia.

Taken together, our results indicate that DHA displays cytotoxic activity in colon cancer cells under normoxic and hypoxic conditions.

### Effect of dihydroartemisinin on short-term and long-term cell viability in HCT116 cells

**Figure 3:**
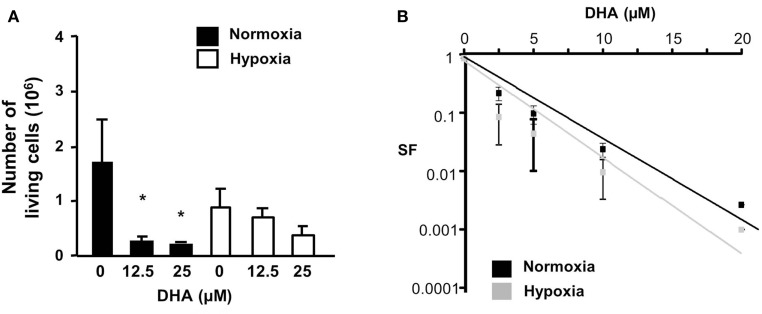
**DHA reduces cell survival and eradicates clonogenic tumor cells in normoxia and hypoxia**. **(A)** HCT116 cell were treated with DHA (12.5, 25 μM) or with ethanol (solvent control, 0 μM DHA). Forty-eight hours later, living cells were counted using trypan blue exclusion dye. *P*-values were calculated applying unpaired two-tailed *t*-test; *P < 0.05. DHA significantly reduced the number of viable cells under normoxic conditions, but only slightly decreased the number of viable cells under hypoxic conditions. **(B)** HCT116 cells were plated for colony formation assay, treated 24 h later for 24 h with 0–20 μM DHA under normoxic (21% O_2_) or hypoxic conditions (0.2% O_2_) and subsequently further incubated at 21% O_2_ for additional 10 days. Values were normalized to the plating efficiency of the cells treated with ethanol (0 μM DHA). Data show the surviving fractions (SF) from three independent experiments (means ± SD). DHA-induced eradication of clonogenic tumor cells was slightly better under hypoxic than under normoxic conditions.

Although DHA induced cell death in colon cancer cells, the drug also might affect the cell proliferation rate. Thus, we analyzed the amount of viable HCT116 cells in a short-term assay measuring the number of viable cells 48 h after treatment with DHA (Figure 3A) as well as in a long-term assay measuring the surviving fraction in response to DHA (Figure 3B) under normoxic or hypoxic conditions. Under normoxia, the number of living cells was significantly reduced 48 h after treatment with 12.5 or 25 μM DHA. In hypoxia, the number of living cells was already slightly decreased without treatment and was further lowered by treatment with DHA, however, not to the same extent as under normoxic conditions. On the other hand, the long-term assay showed that the number of surviving cells able to regrow and form a colony declines with increasing DHA concentration. Surprisingly, DHA treatment reduced the surviving fraction even slightly more efficiently under hypoxic conditions when compared to normoxic conditions. Our results indicate that the long-term toxicity of DHA is slightly improved under hypoxia as compared to normoxia, though the number of surviving cells was slightly higher after short-term treatment under hypoxic conditions. The results suggest that DHA exerts pronounced long-term anti-neoplastic effects that enhance clonogenic cell death particularly under hypoxic conditions.

### Deprivation of oxygen results in a switch from caspase-dependent apoptosis to caspase-independent apoptosis in response to dihydroartemisinin

**Figure 4:**
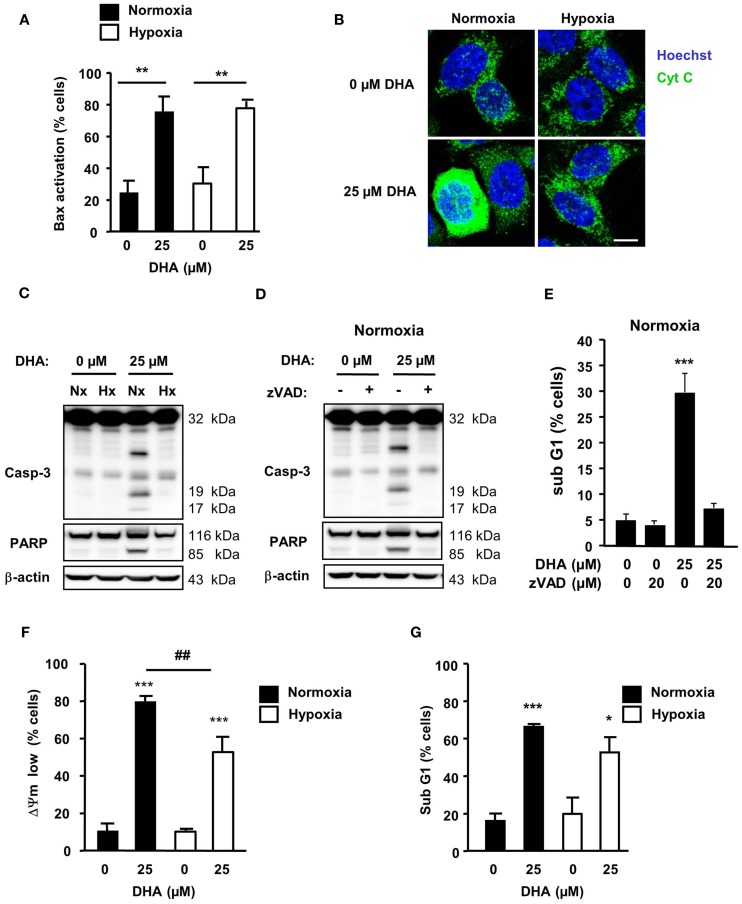
**DHA induces caspase-dependent apoptosis in normoxia but a caspase-independent apoptotic cell death in hypoxia**. HCT116 cells were treated for 12–48 h under normoxic and hypoxic conditions with solvent control (0 μM), 12.5 or 25 μM DHA as indicated. **(A)** DHA-induced Bax-activation is similar in normoxia and hypoxia. Bax-activation was monitored by flow cytometry 12 h after treatment using an activation-specific anti-Bax antibody. **(B)** DHA induces significant release of cytochrome *c* only in normoxia. Cytochrome *c* release was visualized 12 h after DHA treatment by immunofluorescence (Zeiss Cell Observer fluorescence microscope with Apotome; Axiocam MRm camera; GFP filter). Scale bar corresponds to 10 μm. Data show representative pictures from three independent experiments. **(C,D)** Activation of caspase-3 and cleavage of caspase-3 substrate PARP were analyzed by western blotting at 24 h after treatment with DHA. Data are representative blots out of three independent experiments. **(C)** DHA-induced caspase-activation is abrogated in hypoxia. Caspase-3 activation **(D)** and DNA-fragmentation **(E)** can be blocked by co-treatment with the pan-caspase inhibitor zVAD-fmk under normoxic conditions. DHA induces depolarization of the mitochondrial membrane potential [ΔΨm, **(F)**] and DNA-fragmentation [sub-G1, **(G)**] in normoxia and hypoxia. Depolarization of ΔΨm **(F)** and DNA-fragmentation **(G)** were determined at 24 h after treatment by flow cytometry. **(A,E–G)** Data represent means ± SD from at least three independent experiments. *P*-values were calculated applying unpaired two-tailed *t*-test; *P < 0.05; **^, ##^*P* < 0.01; ***P < 0.001. *Indicates the significance between the solvent-treated control cells and DHA-treated cells either in normoxia or hypoxia, ^#^indicates the significance between normoxic and hypoxic cells treated with the same concentration of DHA.

Earlier investigations in Jurkat T-lymphoma cells had demonstrated that, under standard normoxic culture conditions, DHA activates the intrinsic apoptosis pathway to trigger ROS-dependent cell death (11). Intrinsic apoptosis is characterized by the activation of the Bcl-2 effector proteins Bax and/or Bak which, in turn, induce the permeabilization of the outer mitochondrial membrane and cytochrome *c* release from the mitochondrial intermembrane space into the cytosol, which acts as a co-factor to activate the caspase cascade [for review see Ref. (18)]. This ultimately results in dissipation of the ΔΨm and DNA-fragmentation. To gain insight into the regulation of DHA-induced cell death in HCT116 cells under normoxic and hypoxic conditions, we compared the canonical steps of the intrinsic apoptosis pathway in HCT116 cells in response to DHA at 21 and 0.2% O_2_ (Figure 4). In normoxia, DHA readily induced Bax-activation (Figure 4A), cytochrome *c* release into the cytosol (Figure 4B), and cleavage of caspase-3 and of the caspase-3 substrate PARP (Figure 4C), dissipation of ΔΨm (Figure 4F), and DNA-fragmentation (Figure 4G), respectively. Though Bax-activation was detected to a similar extent upon DHA treatment under hypoxic conditions, cytochrome *c* release into the cytosol, caspase-3 activation, and PARP-cleavage were abrogated under these conditions. In addition, blocking caspase-activation by co-treatment with pan-caspase inhibitor zVAD-fmk and DHA under normoxia prevented cleavage of caspase-3 and PARP (Figure 4D) as well as DNA-fragmentation (Figure 4E), suggesting that the canonical apoptosis pathway is activated in response to treatment with DHA under normoxic conditions.

Interestingly, mitochondrial dissipation (Figure 4F) and DNA-fragmentation (Figure 4G) were only slightly reduced in hypoxic cells in response to treatment with 25 μM DHA when compared to treatment under normoxic conditions. Thus, our results suggest that, in response to DHA, a caspase-independent apoptotic cell death occurred when cells were treated under hypoxic conditions.

### Dihydroartemisinin-induced ROS production contributes to the cytotoxic effects under normoxic and hypoxic conditions

**Figure 5:**
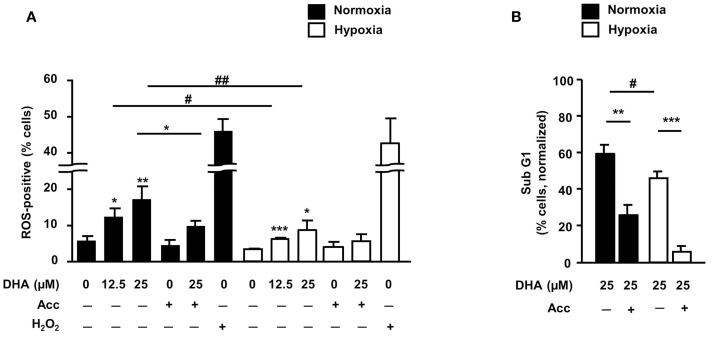
**ROS are important mediators of DHA-induced cytotoxicity in both, normoxia and hypoxia**. **(A,B)** HCT116 cells were pre-incubated for 30 min in the absence or presence of the radical scavenger *N*-acetylcystein (Acc; 2 mM) and then treated for 24 h **(A)** or 48 h **(B)** with DHA (0–25 μM) under normoxic and hypoxic conditions as indicated. **(A)** DHA induces ROS under normoxic and hypoxic conditions in a concentration-dependent manner. Treatment with 250 μM H_2_O_2_ for 2 h was applied as a positive control for ROS formation. Pre-treatment with the ROS scavenger *N*-acetylcystein (Acc) was used to intercept and neutralize ROS. ROS formation was detected by flow cytometry upon loading of the cells with the oxidation-sensitive dye DHE. **(B)** DHA-induced DNA-fragmentation is ROS-dependent in normoxia and hypoxia. DNA-fragmentation (sub-G1) was analyzed by flow cytometry after staining the cells with PI. Data represent means ± SD from three independent experiments. *P*-values were calculated applying unpaired two-tailed *t*-test. *^, #^*P* < 0.05; **^, ##^*P* < 0.01; ***P < 0.001. *Indicates the significance between the solvent-treated control cells and DHA-treated cells either in normoxia or hypoxia, ^#^indicates the significance between normoxic and hypoxic cells treated with the same concentration of DHA.

Earlier reports including own data pointed to ROS-dependent induction of apoptosis by DHA and related compounds (11, 15, 16). Therefore, we next examined whether treatment with DHA would increase cellular ROS levels in HCT116 cells and whether DHA-induced apoptosis was ROS-dependent. As shown in Figure 5A, the amount of ROS-positive cells increased in response to treatment with DHA in a concentration-dependent manner under both, normoxic and hypoxic conditions. However, ROS production was significantly lower when treatment was performed in severe hypoxia. Moreover, pre-treatment with the radical scavenger Acc not only decreased DHA-induced ROS production (Figure 5A), but concurrently decreased DHA-induced DNA-fragmentation under normoxic and hypoxic conditions (Figure 5B). These observations demonstrate that the induction of ROS is essential for the cytotoxic action of DHA in both treatment conditions.

### Normoxic and hypoxic HCT116 cells up-regulate different sets of BH3-only proteins under normoxic and hypoxic conditions

**Figure 6:**
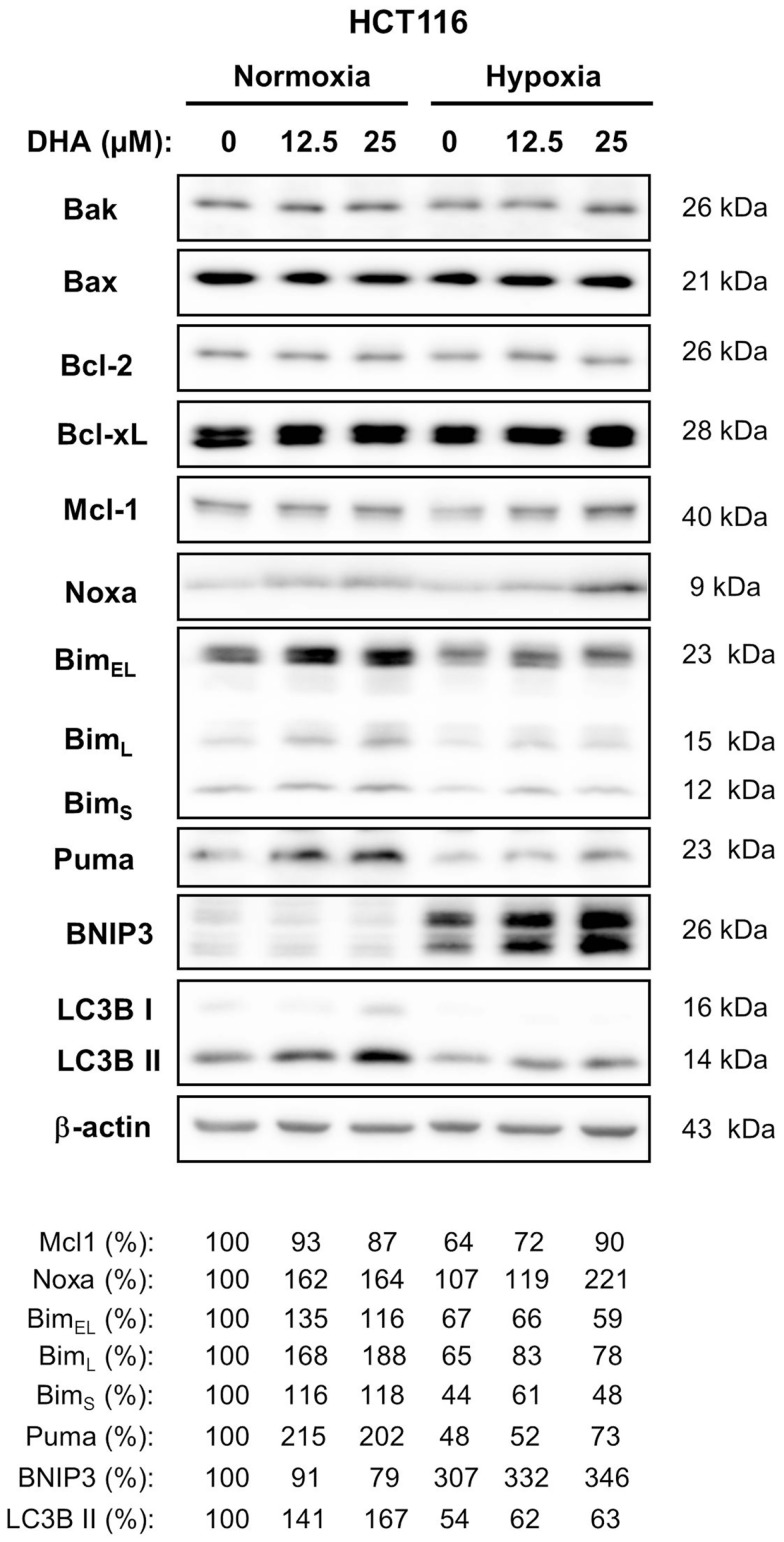
**BH3-only proteins Bim, Puma, Noxa, and BNIP3 are differentially regulated in response DHA in normoxia and hypoxia**. HCT116 cells were treated with 0, 12.5, or 25 μM DHA under normoxic or under hypoxic conditions as indicated. Twenty-four hours later, whole cell lysates were made. Levels of anti-apoptotic and pro-apoptotic proteins of the Bcl-2 family were analyzed by western blot. Induction of autophagy was accessed by western blot using an antibody against LC3B and a subsequent analysis of processed LC3B form (LC3B II, 14 kDa). Levels of anti-apoptotic Bcl-2 and Bcl-xL and pro-apoptotic Bax and Bak did not change in response to treatment with DHA under normoxic and hypoxic conditions. Levels of BH3-only protein Noxa were increased in response to DHA in normoxia as well as in hypoxia. Levels of Bim and Puma were only increased by DHA in normoxia, whereas levels of BNIP3 were elevated by DHA only in hypoxia. Induction of autophagy in response to DHA was observed only under normoxic conditions. Protein levels were analyzed by densitometry and normalized to β-actin levels. Relative protein levels are shown below the respective blots. Data show representative blots from at least two independent experiments.

Since activation of Bax seems to be an important step during DHA-induced cell death under normoxic and hypoxic conditions, a western blot analysis was performed to examine whether a change in the expression of other Bcl-2 family members could contribute to apoptosis induction (Figure 6). HCT116 cells express higher levels of Bax than the closely related Bak as well as higher levels of the anti-apoptotic Bcl-xL than the closely related protective Bcl-2. However, the expression of these four proteins was not affected by DHA. In contrast, treatment with DHA resulted in slightly increased levels of the anti-apoptotic Mcl-1, although Mcl-1 levels were lower when cells were cultured in hypoxic condition as compared to normoxic conditions. Interestingly, the pro-apoptotic BH3-only protein Noxa showed similar regulation like its interacting partner Mcl-1.

In contrast, levels of the BH3-only proteins Bim and Puma were only elevated in response to treatment with DHA when treatment was performed under normoxic conditions. Instead, expression of the BH3-only protein BNIP3 was up-regulated when HCT116 cells were exposed to severe hypoxia and its levels further increased after DHA treatment though BNIP3 was not detected in normoxia.

Taken together, our data suggest that different sets of BH3-only proteins are up-regulated in response to DHA under normoxic and hypoxic conditions.

BNIP3 is known to interact with Bcl-2 thereby counteracting Bcl-2’s anti-apoptotic activity or preventing Bcl-2 from binding to Beclin-1. The former interaction results in apoptosis induction whereas the latter one results in autophagy induction. In addition, autophagy-associated cell death might also contribute to clonogenic cell death. Therefore, we examined the induction of autophagy by analyzing LC3B processing to a 14 kDa form in HCT116 cells. Interestingly, an increase of the 14 kDa LC3B band was observed under normoxic conditions when BNIP3 expression was hardly detectable. In contrast, under hypoxic conditions, no increase of processed LC3B could be detected although BNIP3 levels were dramatically increased. Thus, our results suggest that BNIP3 does not regulate autophagy but rather apoptosis in hypoxic HCT116 cells.

## Discussion

Hypoxia is a major biological factor that limits the success of anti-cancer treatment. Here, we demonstrate for the first time that the anti-neoplastic cyclic endoperoxide DHA is a hypoxia-active anti-cancer drug that efficiently eradicates colon cancer cells not only under normoxic conditions but also when treatment is performed in a severely hypoxic microenvironment.

### Anti-neoplastic activity in normoxia and hypoxia

Under normoxic conditions, DHA potently induced cell death, particularly apoptosis, in colon cancer cells whereas necrotic cell death was only induced to a substantial extent when rather high drug concentrations were used.

The main mode of DHA-induced cell death in colon cancer cells under hypoxic conditions also resembled apoptosis. This assumption is based on the findings that dissipation of the mitochondrial membrane potential as well as DNA-fragmentation were still observed in severe hypoxia, though caspase-3 activation was greatly reduced. This is reminiscent of a caspase-independent apoptosis with DNA-fragmentation that was described before (19). In this context, previous publications suggested a translocation of apoptosis-inducing factor (AIF) and endonuclease G (EndoG) from mitochondria to the nucleus, where both proteins are able to initiate DNA-degradation independent of caspase-activation (20, 21). The pronounced sensitivity to DHA-induced apoptosis observed in short-term assays strongly correlated with efficient eradication of clonogenic HCT116 cells in long-term colony formation assays. Notably, the drug displayed even slightly higher activity in HCT116 cells treated under severely hypoxic conditions.

### Potential of DHA in anti-cancer therapies

A hypoxic microenvironment renders cancer cells resistant to most standard anti-cancer therapies and, thus, constitutes a major obstacle in the treatment of cancer patients (2). The molecular mechanisms leading to the increased resistance of hypoxic cancer cells to standard chemotherapeutic drugs or ionizing radiation are not fully understood. However, previous publications have demonstrated that hypoxic tumor cells stabilize the hypoxia-inducing factor-1α (HIF-1α) leading to the HIF-1α-dependent cell protection, increased transcription of vascular endothelial factor (VEGF), and tumor vessel formation (3, 22). There is increasing evidence that DHA interferes with HIF-1α activation, VEGF expression, and angiogenesis. On the one hand, DHA treatment reduced hypoxia-induced HIF-1α activation and VEGF expression in multiple myeloma and C6 rat glioma cells resulting in reduced tumor cell growth and angiogenesis, respectively (23, 24). On the other hand, DHA enhanced the toxicity of cisplatinum in lung adenocarcinoma cells *in vivo* and this effect was accompanied by reduced expression of HIF-1α and VEGF and reduced tumor microvessel density (25). Interestingly, this anti-angiogenic effect of DHA was attributed to growth inhibition of endothelial cells and depended on the level of tissue oxygenation and the drug concentration (26). Thus, the anti-angiogenic effects described above differ from our findings where DHA was almost similarly active under normoxic and hypoxic conditions in all three colon cancer cell lines examined (see Figure 1). The pronounced activity of DHA in severe hypoxia suggests that the drug has a great advantage over many standard anti-cancer drugs and radiotherapy whose cytotoxic effects strongly rely on adequate oxygen tensions as well as on intact cell death pathways (7, 27).

Moreover, although not yet examined in clinical trials, DHA is able to improve treatment efficacy in combination with ionizing radiation or cisplatin in preclinical models (25, 28, 29) and thus shows great potential as anti-neoplastic drug.

Of note, drug concentrations used in the present study correspond to DHA plasma levels achievable in humans. Upon repeated intravenous application of 8 mg/kg artesunate, peak plasma concentrations up to 5.8 mg/l (around 20 μM) of its principal active metabolite DHA were measured in healthy volunteers (30).

### Generation of ROS in response to DHA

Up to now, preclinical studies about the anti-neoplastic activity of artemisinin and derivatives had been mostly restricted to experiments conducted in normoxia. These studies demonstrated that the cytotoxic action of these compounds involves the formation of ROS and membrane oxidation upstream of apoptosis-associated changes in mitochondrial function (11, 31, 32). Moreover, chronic treatment of LN-229 glioblastoma cells with artesunate led to a continuous increase in ROS and in oxidative DNA-damage resulting in continuously increasing DNA double-strand breaks and finally tumor cell death (16).

Our present data show for the first time that DHA can induce ROS formation in hypoxia, though at slightly reduced levels. The generated ROS were important mediators of DHA-induced cytotoxicity under normoxic as well as under hypoxic conditions since blocking ROS formation by Acc resulted in abrogation cell death under normoxic or hypoxic conditions. Although DHA-induced oxidative DNA-damage and the resulting DNA-damage response have not been studied here, it is highly likely that treatment with DHA under normoxic as well as under hypoxic conditions will also result in the generation of DNA double-strand breaks and, finally, in cell death due to the inability of the cells to repair the damaged DNA.

Taken together, our novel data indicate that the generation of ROS is a hallmark of DHA-induced cell death under normoxic as well as under hypoxic conditions.

### Role of Bcl-2 family members for the regulation of DHA-induced clonogenic death in hypoxia

The effector proteins of the Bcl-2 protein family, Bax and Bak, are essential for apoptosis induction. Both of them become activated in response to many apoptotic stimuli (11, 33–35). In addition, up-regulation of Bax levels was detected in response to cerebral ischemia or following exposure to ionizing radiation in a p53-dependent way (36–38). Although the levels of pro-apoptotic proteins Bax and Bak were not changed in response to treatment with DHA, their activation might be essential for DHA-induced cell death. Indeed, Bax was activated in most HCT116 cells after treatment with DHA under normoxia and hypoxia. Thus, the results suggest, that at least Bax might be a very important mediator of DHA-induced cytotoxicity under normoxic as well as under hypoxic conditions.

Furthermore, anti-apoptotic proteins of the Bcl-2 family participate in the regulation of the balance between apoptosis and autophagy in response to cell stress, including hypoxia (39, 40). They associate either with Bax or Bak to prevent outer mitochondrial membrane permeabilization and apoptosis induction or with Beclin-1 preventing this molecule from induction of autophagy (18, 41–45). Both, apoptosis and autophagy could contribute to clonogenic cell death induced by DHA. However, Bcl-2 or Bcl-xL levels did not change in response to DHA treatment under normoxic or hypoxic conditions. Only Mcl-1 levels were decreased when cells were grown under hypoxia. Treatment with DHA slightly increased Mcl-1 levels under hypoxia, but had no effect under normoxia. Yet, the three protective proteins can be released from a complex formed with Bax/Bak or Beclin-1 by a competitive interaction with BH3-only proteins such as Bim, Puma, Noxa, and BNIP3. Interestingly, Noxa levels were increased in response to DHA under normoxia as well as hypoxia. In contrast, Bim and Puma levels were up-regulated only under normoxic conditions, whereas BNIP3 levels were increased only under hypoxic conditions after treatment with DHA. Furthermore, previous publications have shown that BNIP3 up-regulation was observed preferentially in hypoxic cells and was often associated with autophagy induction due to the release of Beclin-1 from interaction with Bcl-2 or Bcl-xL by replacement (46). However, BNIP3 is also able to induce apoptosis by binding to Bcl-2 and Bcl-xL (47). Surprisingly, autophagy induction as measured by LC3B processing was detected only in normoxic cells in response to DHA treatment in the absence of BNIP3. In contrast, under hypoxia, when BNIP3 levels were greatly increased, processing of LC3B could not be detected. Thus, our data suggest that BNIP3 regulates rather apoptosis than autophagy under hypoxic conditions. However, up-regulation of the BH3-only proteins, Bim and Puma could result in an enhanced interaction with Bcl-2 and Bcl-xL to induce both, autophagy and apoptosis, under normoxic conditions. Taken together, our data clearly demonstrate a differential expression of BH3-only proteins under normoxic and hypoxic conditions in response to treatment with DHA in HCT116 colon cancer cells to induce cancer cell death.

Given that we did not analyze the protein complexes in more detail, we cannot state whether the discussed BH3-only proteins associate with the anti-apoptotic Bcl-2 members to release Beclin-1 and induce autophagy or to activate Bax and Bak to induce apoptosis. A further analysis of Bcl-2, Bcl-xL, and Mcl-1 as well as their interacting partners will clarify the molecular mechanisms by which DHA induces apoptosis and autophagy under normoxic and hypoxic conditions.

## Conclusion

In contrast to many genotoxic drugs and radiotherapy, which are generally less efficient in hypoxic tumor cells, DHA exerts pronounced anti-neoplastic effects under severely hypoxic conditions. While the canonical intrinsic apoptosis pathway seemed to be predominantly activated by DHA in oxygenated cells, DHA induced a caspase-independent apoptosis-like cell death in severe hypoxia. Since DHA targets normoxic as well as hypoxic cells with equal potency, the drug might be a promising tool to improve treatment outcome, particularly in hypoxic human tumors resistant to conventional therapies.

## Author Contributions

Verena Jendrossek designed the study. Teona Ontikatze, Justine Rudner, and René Handrick performed the experiments and analyzed the data. Justine Rudner and Verena Jendrossek interpreted the data, drafted and revised the manuscript. All authors read the manuscript and gave the final approval for publication. The authors agree to be accountable for all aspects of the work in ensuring that questions related to the accuracy or integrity of any part of the work are appropriately investigated and resolved.

## Conflict of Interest Statement

The authors declare that the research was conducted in the absence of any commercial or financial relationships that could be construed as a potential conflict of interest.

## Abbreviations

Acc, *N*-acetylcysteine; CCCP, protonophore carbonyl cyanide m-chlorophenylhydrazone; DHA, dihydroartemisinin; ΔΨm, mitochondrial transmembrane potential; Hx, hypoxia; LC3B, light chain 3B; Nx, normoxia; PARP, poly (ADP-ribose) polymerase; PE, plating efficiency; ROS, reactive oxygen species; TMRE, tetramethylrhodamine ethyl ester perchlorate; zVAD-fmk, benzyloxycarbonyl-Val-Ala-Asp(OMe)-fluoromethylketone.
